# Seven Years of Monitoring Susceptibility to Cry1Ab and Cry1F in Asian Corn Borer

**DOI:** 10.3390/toxins15020137

**Published:** 2023-02-07

**Authors:** Yueqin Wang, Wenlu Zhao, Shuang Han, Lianxia Wang, Xue Chang, Kaiqiang Liu, Yudong Quan, Zhenying Wang, Kanglai He

**Affiliations:** 1 State Key Laboratory for the Biology of the Plant Diseases and Insect Pests, Institute of Plant Protection, Chinese Academy of Agricultural Sciences, Beijing 100193, China; 2 Dezhou Academy of Agricultural Sciences, Dezhou 253000, China; 3 Qiqihar Sub-Academy of Heilongjiang Academy of Agricultural Sciences, Qiqihar 161006, China; 4 Institute of Plant Protection, Jilin Academy of Agricultural Sciences, Gongzhuling 136100, China

**Keywords:** *Ostrinia furnacalis*, Bt toxins, monitoring, susceptibility

## Abstract

Resistance monitoring in the Asian corn borer, *Ostrinia furnacalis*, is necessary to accommodate the commercial introduction and stewardship of Bt maize in China. The susceptibility of 56 *O. furnacalis* field populations, collected between 2015 and 2021 from the corn belt regions of China, to Cry1Ab and Cry1F toxins was determined. Neonate larvae (within 12 h after hatching) were placed on the surface of semi-artificial agar-free diet incorporating a series of concentrations of purified toxins, and mortality was evaluated after 7d. The median lethal concentration (LC_50_) values of Cry1Ab and Cry1F were 0.05 to 0.37 µg/g (protein/diet) and 0.10 to 1.22 µg/g, respectively. Although interpopulation variation in susceptibility to the toxins was observed, the magnitude of the differences was 5.8-fold and 8.3-fold for Cry1Ab and Cry1F, respectively. These results suggested that the observed susceptibility differences reflect natural geographical variation in response and not variation caused by prior exposure to selection pressures. Therefore, the *O. furnacalis* populations were apparently still susceptible to Cry1Ab and Cry1F across their range within China. The monitoring data established here will serve as a comparative reference for early warning signs of field-evolved resistance after the cultivation of Bt maize in China.

## 1. Introduction

Crops genetically modified to express insecticidal proteins encoded by genes from the bacterium *Bacillus thuringiensis* (i.e., Bt) for the control of important lepidopteran pests (such as *Ostrinia nubilalis*, *Spodoptera frugiperda*, and *Helicoverpa zea*) have been widely planted all over the world since first commercialized in 1996 [1]. In 2021, the area planted to insect-resistant Bt crops, alone or stacked with other traits such as herbicide tolerance or drought tolerance, exceeded more than 100 million ha in over 20 countries distributed across six continents [1]. Benefits associated with the adoption of Bt crops include pest suppression, increased or stabilized yields, decreased application of chemical insecticides that were more harmful to the environment, no adverse effect on beneficial insects, and, in the case of Bt corn, reduced the incidence of toxic fungal compounds by reducing insect damage that made the corn more susceptible to the fungi [2,3,4,5].

However, some of the economic and environmental benefits of Bt crops would be limited by the evolution of Bt resistance within the target pest populations, particularly with respect to the earlier commercialized single-trait products. In fact, field-evolved practical resistance, which has reduced the number of Bt toxins in transgenic crops that are effective against some major pests to two, one, or none, to Bt corn, has been documented in several species, including *Busseola fusca* and *S. frugiperda* in *cry1Ab* corn, *S. frugiperda* and *Striacosta albicosta* in *cry1F* corn, and *Diatraea saccharalis* in *cry1A.105* corn [6,7,8,9,10]. As the field-resistant populations reduce their susceptibility to the Bt proteins produced by Bt crops, it is necessary to alter the current pest management practices. In order to make up for the loss of efficacy of Bt traits, farmers may increase their use of chemical insecticides that are more harmful to the environment and non-target organisms and ultimately alter cultivation systems, which may increase farm economic costs and break the sustainability of the farming system [11]. Therefore, developing and implementing science-based insect resistance management strategies is necessary to manage the resistance of target insect pests to Bt crops and preserve the long-term effectiveness of Bt crops. Besides, insect resistance management strategies have been an essential component of development and a regulatory requirement for registration of Bt events [12].

The insect resistance management strategies for Bt crops are dependent on the development of effective resistance monitoring programs that are able to detect early warning signs at an early stage, which will offer valuable information to trigger alternative management decisions in a timely manner [13]. The development of appropriate bioassay methods and the establishment of the baseline susceptibility among field populations across the geographic range of the target species are the initial steps in developing such a monitoring program. With this information, the potential changes in population susceptibility to specific Bt toxins can be identified [13]. Data generated through resistance monitoring also allows registrants, agribusinesses, risk assessors, and regulators to assess whether the current resistance management tactics manage Bt resistance in the target insect pest populations adequately and efficiently [11]. Moreover, the refuge requirement is the critical factor contributing to the success of the refuge-high dose strategy, and resistance monitoring activities are also tailored to collect information on the presentation of refuges in non-Bt host plants, Bt crop cultivation levels, and applied pest management practices [11]. The monitoring data can also be used to select a diagnostic or discriminating dose that can kill 99% of susceptible individuals for resistance monitoring. A diagnostic dose bioassay offers the advantage of being more efficient for detecting low frequencies of resistance alleles, as all individuals are tested at an appropriate dose and none are wasted. Additionally, this method allows for the screening of a great number of individuals and is less labor-intensive than traditional concentration-mortality testing [14,15,16].

Asian corn borer (ACB), *O. furnacalis* (Guenée) (Lepidoptera: Crambidae), is one of the most destructive pests of corn in China, Japan, and countries of Southeast Asia, including Vietnam and the Philippines, as well as Australasia and the Pacific [17]. It can cause considerable yield losses that have been previously reported to be in the range of 10% to 80% and pose a major threat to food security [18,19]. In the Philippines, single trait event MON810 (expressing Cry1Ab protein) commercialization started in 2003, following the strict regulations set by the Department of Agriculture for biotech cultivation approved and signed in 2002 [19]. In 2015, the Ministry of Natural Resources and Environment approved Syngenta’s Bt11 (expressing Cry1Ab protein) corn for commercial cultivation in Vietnam [20]. In China, multiple Bt insect-resistant lines, including DBN9936 (*cry1Ab*), DBN9501 (*cry1Ab*), Ruifeng125 (*cry1Ab* and *cry2Aj*), have received biosafety certificates authorized by the Ministry of Agriculture and Rural Affairs of the People’s Republic of China since 2019 (http://www.moa.gov.cn/ztzl/zjyqwgz/spxx/201912/P020200121588032-501444.pdf, accessed on 2 December 2019). Laboratory bioassays and field trials indicated that the three transgenic Bt maize events were highly toxic to ACB and could provide season-long protection against ACB [21,22]. And now, the central government is truly ready for large-scale commercialization of this genetically engineered (GE) crop. Besides, these GE crops were already illegally cultivated in the major corn-growing areas of China.

The objective of the current study was to monitor the susceptibility of *O. furnacalis* to purified Cry1Ab and Cry1F collected from geographically distinct sites across the North Spring Corn Region and Huang-Huai-Hai (Yellow, Huai, and Hai River) Summer Corn Regions, which make up about 75% of the total corn planting area [23]. The susceptibility data established here will serve as a baseline for future resistance monitoring work and may provide information that will allow the development of a diagnostic or discriminating dose that will kill 99% of susceptible individuals that would be more efficient in the detection of resistant populations after the cultivation of Bt maize in China.

## 2. Results

**Figure 1:**
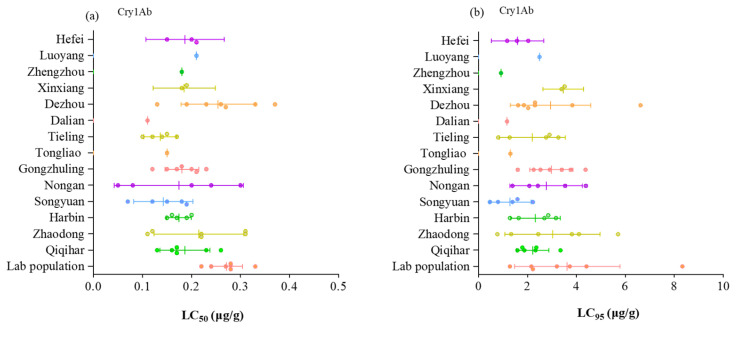

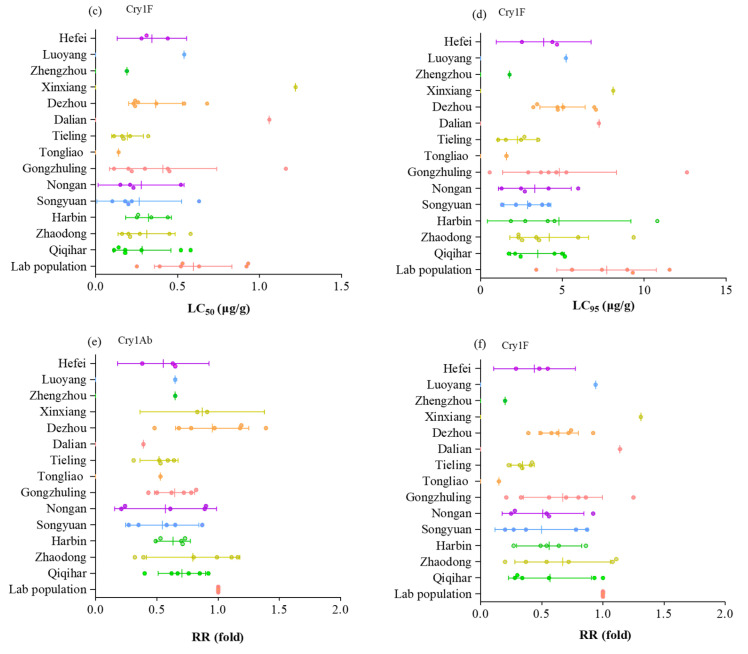
Susceptibility of *O. furnacalis* neonate larvae to Cry1Ab and Cry1F toxins for the laboratory population and field populations collected in major corn-growing regions from 2015–2021. (**a**) LC_50_ values of Cry1Ab to the laboratory population and field populations; (**b**) LC_95_ values of Cry1Ab to the laboratory population and field populations; (**c**) LC_50_ values of Cry1F to the laboratory population and field populations; (**d**) LC_95_ values of Cry1F to the laboratory population and field populations; (**e**) RR values of Cry1Ab to the laboratory population and field populations at the LC50 level; (**f**) RR values of Cry1F to the laboratory population and field populations at the LC_50_ level. Each dot represents one year’s monitoring result.

The susceptibility of neonate Asian corn borer larvae to purified Cry1Ab and Cry1F toxins for the laboratory population and field populations are presented in Tables S1 and S2, and Figure 1. The LC_50_ values of Cry1Ab ranged from 0.05 µg/g (protein/diet) (2019, Nongan) to 0.37 µg/g (2017, Dezhou), and the LC_95_ values ranged from 0.46 µg/g (2015, Songyuan) to 6.62 µg/g (2017, Dezhou). LC_50_ and LC_95_ values of Cry1F ranged from 0.10 µg/g (2016, Songyuan) to 1.22 µg/g (2015, Xinxiang) and from 0.55 µg/g (2019, Gongzhuling) to 12.62 µg/g (2015, Gongzhuling). As changes in the source of toxins can significantly impact the LC_50_ and LC_95_ values, we used the resistance ratio (RR) to track the resistance of ACB in fields. Based on the RR, the differences between the most tolerant and the most susceptible populations were 5.8-fold and 8.3-fold for Cry 1Ab and Cry1F at the LC_50_ level, respectively. Based on the criterion of non-overlap of 95% fiducial limits in LC_50_s, significant differences in susceptibility were detected among some of the populations involved.

**Figure 2:**
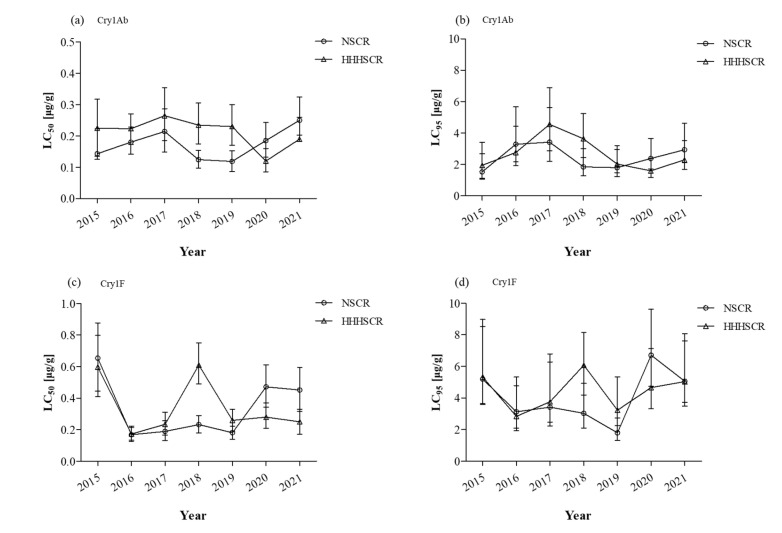
Susceptibility of *O. furnacalis* neonate larvae to Cry1Ab and Cry1F toxins in the corn ecological regions from 2015–2021. NSCR: North Spring Corn Region; HHHSCR: Huang-Huai-Hai Summer Corn Region. (**a**) LC_50_ values of Cry1Ab to NSCR and HHHSCR populations; (**b**) LC_95_ values of Cry1Ab to NSCR and HHHSCR populations; (**c**) LC_50_ values of Cry1F to NSCR and HHHSCR populations; (**d**) LC_95_ values of Cry1F to NSCR and HHHSCR populations.

The monitoring results for the corn ecological regions North Spring Corn Region and Huang-Huai-Hai Summer Corn Region are presented in Figure 2. The susceptibility of Cry1Ab to field populations of *O. furnacalis* collected from the North Spring Corn Region and expressed as LC_50_ values ranged from 0.12 µg/g (2019) to 0.25 µg/g (2021). When expressed as LC_95_, values ranged from 1.53 µg/g (2015) to 3.42 µg/g (2017). LC_50_ and LC_95_ values of Cry1F ranged from 0.17 µg/g (2016) to 0.65 µg/g (2015) and from 1.79 µg/g (2019) to 6.71 µg/g (2020). For populations collected from the Huang-Huai-Hai Summer Corn Region, LC_50_ and LC_95_ values of Cry1Ab ranged from 0.12 µg/g (2020) to 0.27 µg/g (2017) and from 1.59 µg/g (2020) to 4.55 µg/g (2017), respectively; LC_50_ and LC_95_ values of Cry1F ranged from 0.17 µg/g (2016) to 0.61 µg/g (2018) and from 2.84 µg/g (2020) to 6.08 µg/g, respectively. In general, the LC_50_ values of the Huang-Huai-Hai Summer Corn Region field populations were greater than those of the North Spring Corn Region.

## 3. Discussion

It is essential to determine the sensitivity of the target insects to Bt toxins before the commercial release of transgenic maize that expresses Bt toxins, and monitoring data is a necessary component of resistance management strategies. In the present study, the susceptibility of ACB field populations collected from different provinces and regions to the Bt toxins Cry1Ab and Cry1F was monitored between 2015 and 2021. These data will serve as a comparative reference for early detection of field-evolved resistance after the deployment of Bt maize in China.

Based on the concentration-mortality responses, the highest RRs for Cry1Ab and Cry1F were 1.4 (0.9–2.1) (Dezhou, 2017) and 1.3 (0.9–1.9) (Xinxiang, 2015) at the LC_50_ level, respectively. The two field populations were less sensitive to Cry1Ab and Cry1F compared to other populations, probably because the Bt cotton planting area in these two regions occupied a relatively large proportion of this area. Cotton is another host plant for ACB, and transgenic cotton that expresses Bt toxins likely selected the insect intensively for resistance. The RR, typically calculated as the LC_50_ for a resistant population divided by the LC_50_ for a susceptible population, reflected the magnitude of resistance. Tabashnik et al. [24] define RR values ≥ 10 as the development of insect resistance to insecticides. Therefore, the field populations in this study appeared susceptible to the toxins because none of them reached that level. All the tested populations showed different susceptibilities to Cry1 toxins but were still within the range of natural tolerance. And the reported difference in susceptibility may be due to natural variation among the geographically distinct populations and agroecological conditions [25]. In general, some field populations were more susceptible than the reference colony under long-term rearing in the laboratory. These dissimilarities were possibly related to fitness.

As ACB is one of the most destructive insect pests of corn throughout Asia and Southeast Asia, the baseline susceptibility of different geographic populations of ACB to Cry1Ab or Cry1A.105 has been estimated in several countries, including China, Vietnam, and the Philippines [19,20,25,26]. In 2005, the baseline susceptibility to Cry1Ab of ACB from the corn belt across the northeast and east-central parts of China was first reported [25]. Geographically distinct ACB populations were highly susceptible to Cry1Ab in China and Vietnam, and the Cry1Ab resistance allele frequency was as low as 0.0048 in China [20,26]. In the Philippines, MON89034, a pyramided transgenic corn event expressing Cry1A.105 and Cry2Ab2 that were highly effective against ACB, was approved for commercial use in 2010. The interpopulation variation in susceptibility to Cry1A.105 and Cry2Ab2 among populations have been reported to be <10-fold [19].

Although Bt corn is illegally planted in the North Spring Corn Region, the field populations are still highly susceptible to Cry1Ab and Cry1F. This is partly because the plantation area is limited. On the one hand, as the most abundant crop, non-Bt corn that is planted near, adjacent to, or within the Bt corn fields can supply natural refuges for ACB. Rare resistant individuals emerging from Bt corn fields will mate with the relatively abundant susceptible pests that thrive in non-Bt refuges. If the resistance is recessive, the resulting heterozygous offspring will die on Bt maize and thus delay the evolution of resistance [27].

Without exposure to any Bt pesticides and Bt proteins, in general, many factors may contribute to the interpopulation variations, such as the parental populations’ vigor, the generation tested, agronomic hosts, the nutritional status of the egg, and the ecological environment [28,29,30]. Interpopulation variations in susceptibility to Bt toxins may also be related to corn-growing areas. For example, LC_50_ values of the field populations from the Huang-Huai-Hai Summer Corn Region were greater than those of other regions [25,26]. The incidence of biomortality factors, especially the natural infestation by Bt of the ACB, was prevalent in this region. Also, cultivation of transgenic cotton may indirectly contribute to the decreased susceptibility to Cry1Ab and Cry1F in ACB in the Huang-Huai-Hai Summer Corn Region. Differences between preparations associated with different ways of purification, trypsin activation or formulation all seemed to have significant effects on toxicity [31,32,33,34]. There is no evidence to that Bt susceptibility is linked to pheromone race and voltinism ecotype [25].

In the current study, the susceptibility evaluation involved complete dose-response tests requiring a series of doses that produce 10–100% mortality. Such techniques are not efficient for detecting frequencies of resistance as low as 0.005, but they are enough to monitor resistance that has reached high levels [35]. An alternative to traditional concentration-response tests involves the use of diagnostic or discriminating doses, which offer the advantage of being more efficient at detecting low frequencies of resistance and requiring much less time [14]. When diagnostic concentrations were used to monitor resistance to Cry1 toxins, despite some survival in the discriminating dose, it did not indicate that resistance to Cry1 was found. Resistance was defined as ‘a phenotype of an individual that can survive on the transgenic insecticidal plant from egg to adult and produce viable offspring’, that is, survivors that were able to complete one life cycle on Bt corn would be designated resistant to Cry protein. The individuals that survive at the diagnostic concentration must be subjected to additional tests to (1) determine if resistance was inherited among individuals that survived at the diagnostic concentration; (2) quantify the magnitude of resistance; (3) determine survival by exposing survivors to Bt plants or Bt plant tissues; and (4) estimate the frequency of resistance alleles among field populations [24,32]. Populations collected in Minnesota exhibited significantly high survival at the discriminating concentration. However, the resistant colony did not survive when reared on vegetative-stage plant tissues. Compared with the susceptible control strain, the resistant colony showed increased survival and reduced growth rates on reproductive plant tissues such as pollen and silks. Variation in Bt protein expression levels in different tissues during vegetative and reproductive development stages may account for such survival in reproductive-stage plants [32]. In an effort to further increase the sensitivity and precision of the monitoring program, it is necessary to select for resistance to Bt toxins in a laboratory colony of target insect pests, as the resistant strains can provide invaluable tools for resistance monitoring [32].

The commercialization of Bt corn has been approved for testing in pilot experiments by the Chinese government. Once largely commercialized, various tactics must be employed in an attempt to prevent or delay the development of insect resistance, including a monitoring program, the promotion of refuge areas, the introduction of pyramided Bt crops, the application of Integrated Pest Management practices, and also proactive engagement with growers, agribusinesses, and regulators. However, pyramided Bt crops producing Cry1Ab and Cry1F should be avoided, as Cry1Ab and Cry1F can recognize the same receptor protein in ACB midguts [36]. The asymmetrical cross-resistance in ACB between Cry1Ab and Cry1F has been reported [37]. Resistance to the pyramids will evolve faster because one of the toxins in the pyramid acts as a stepping stone for resistance. As the major host plant and the most abundant “natural refuges”, corn is a major factor that contributes to the maintenance of cotton bollworm, *H. armigera*, susceptibility to Cry1Ac protein (produced by the first generation of Bt cotton) since Bt cotton was commercialized in 1997 in China. Once Bt maize is commercialized on a large scale, a key refuge for cotton bollworm will be lost, and resistance to Bt cotton may evolve more rapidly. Undoubtedly, the replacement of the first Bt cotton varieties producing Cry1A with the second generation Bt cotton producing two or more Bt toxins that belong to either the Cry protein family or the vegetative insecticidal protein family could be more durable and lead to substantial delays in the resistance evolution of *H. armigera* in China.

## 4. Conclusions

The present study was carried out to monitor for changes in susceptibility to active Cry1Ab and Cry1F in field populations of *O. furnacalis* that were collected from the North Spring Corn Region and the Huang-Huai-Hai Summer Corn Region. Although the susceptibilities among the populations were different, the field populations were still susceptible to Cry1Ab and Cry1F. Besides, the low geographical differences in the susceptibility to Cry1Ab and Cry1F among populations were attributed to natural variations and were not related to prior exposure to selection pressures. The determination of susceptibility to the Bt toxins in field populations of the target insect pest is important prior to the wide cultivation of Bt maize, and our research is the first step in the establishment of an effective monitoring programme and highly effective insect resistance management for *O. furnacalis* in China.

## 5. Materials and Methods

### 5.1. Field Collections and Laboratory Rearing of O. furnacalis

**Figure 3:**
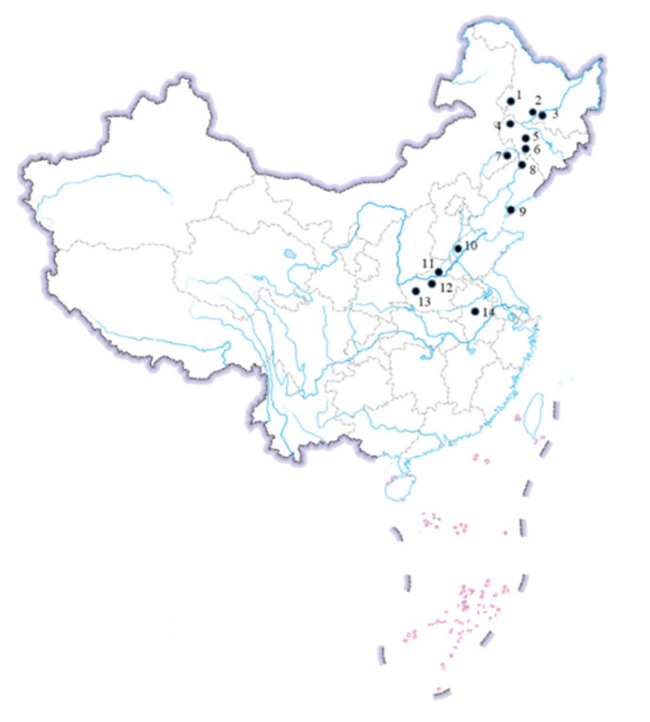
Sampling locations where Asian corn borers were collected.

A total of 56 geographical populations of ACB were collected from seven provinces and regions, including Heilongjiang, Jilin, Liaoning, Inner Mongolia, Shandong, Henan, and Anhui, across the North Spring Corn Region and the Huang-Huai-Hai Summer Corn Region (Figure 3 and Table 1). The sampling locations for collecting ACB had very little use of commercial Bt products in corn. Hence, all monitoring data were tested prior to the widespread cultivation of Bt maize varieties, and any potential selection for Bt resistance alleles was rare. At least 100 individuals of fourth to fifth instar larvae were collected by dissecting damaged maize plants at each sampling location and placed individually in tubes containing fresh plant tissue. After being taken to the laboratory, the larvae were reared to the pupal stage on an artificial diet using standardized rearing techniques [38]. Diapausing fifth instar larvae collected in the autumn were treated with Fangjiangfen (a kind of mild fungicide that is used for body surface treatment of bollworm) to reduce the possible infection of pathogens, especially *Beauveria bassiana* (Bals.) and Bt. The treated larvae were then placed individually into 5-mL tubes (one hole was made in each tube to keep the air circulating) with a piece of plicated paper as a hidden habitat and a piece of wet cotton to supply drinking water [25]. All treated larvae from the same location were packed in a bag and placed outside for 1 to 3 months to terminate diapause, which enabled the larvae to enter post-diapause development and pupate in synchrony [25]. In the next spring, the treated larvae were transferred to 24-well plates with solidified agar. Each well was infected with one larva and a piece of a wet cotton ball (sprayed with water every three days) to supply drinking water. They were then incubated at 27 ± 1 °C, 60–70% RH, and a photoperiod of LD 16:8 h to allow pupation. As the pupation of larvae terminating diapause sometimes did not synchronize very well, one, two, or more generations were necessary to provide a sufficient number of individuals for bioassay.

Adults captured in light traps or sweep nets were transferred to mating cages and covered with waxed paper to provide a substrate for egg laying. Cotton soaked in a solution of 10% honey in water was placed in the mating cages as a food source. Egg masses were collected daily or every two days and allowed to hatch. Neonates hatched from these egg masses were used for subsequent bioassays or used to initiate the next generation if the number was not sufficient for subsequent bioassays. All populations were placed in a 27 ± 1 °C environment with a photoperiod of 16:8 h (L:D) and 80% RH.

The susceptible strain originated from 88 pairs of virgin female and male adults derived from 948 diapause larvae that were collected from corn fields in Shaanxi Province, China, in 2010, where Bt corn was not grown. Their offspring were established as a standard laboratory susceptible strain and were kept alive using artificial diet devoid of Bt protein or pesticides, as well as the rearing techniques described by Song et al. [38].

### 5.2. Bt Toxins

Trypsin-activated Cry1Ab and Cry1F toxins (98% purified crystal proteins) were produced by Marianne P. Carey, Case Western Reserve University, State of Ohio, USA. To prepare the required concentrations, a 50 mM Na_2_CO_3_, pH = 10 buffer solution was used to solubilize these toxins. All samples were analyzed for purity by sodium dodecyl sulfate-polyacrylamide gel electrophoresis (SDS-PAGE), and the concentrations were determined using bovine serum albumin (BSA) as a standard. All toxins were stored at −80 °C until use.

### 5.3. Bioassays

Diet-incorporation bioassays were used to assess the susceptibility of the field populations to Cry1Ab and Cry1F as described by He et al. [25]. Briefly, 10 g of freshly prepared agar-free semi-artificial diet, which was based on soybean flour, corn flour, yeast powder, and sugar, was dispensed into each well of one 48-well bioassay tray (Corning, each well 1.6 mL). An active neonate larva (within 12 h after hatching) was picked up with a soft brush (Penicilli size: L0.9 cm × W0.15 cm, Maries, Shanghai, China) and placed in a well. Bioassay plates were then sealed with a membrane (Cat# 3M-9733, Minnesota Mining and Manufacturing Company, Saint Paul, MN, USA) and placed in a controlled environment as described above. Mortality was determined after 7 days, with mortality recorded as larvae that failed to respond to gentle contact with a fine brush or weighed < 0.1 mg. Bioassays were independently repeated two or three times with a total of 96 or 144 larvae per concentration. The control treatment was with only distilled water, and the maximum accepted mortality in the control was less than 10%.

### 5.4. Data Analysis

Concentration responses of different populations to the two toxins were analyzed by probit regression using Polo Plus V1.0 (LeOra Software Company, Petaluma, CA, USA). The number of larvae tested (n), LC_50_ (lethal concentration for 50% of test organisms) with 95% fiducial limits (FL), LC_95_ with 95% FL, slope with standard errors (Slope ± SE), chi-squared (χ^2^), and resistance ratios (RR) with 95% confidence intervals (CI) were recorded. The RR is the toxin concentration (LC_50_) that kills 50% of the insects tested for a field-derived strain divided by the LC_50_ for a conspecific susceptible strain. Two LC_50_ values were significantly different only if their 95% fiducial limits did not overlap [39].

## Figures and Tables

**Table 1:** Sampling information for the Asian corn borer field populations during 2015–2021.

Province	Field Population	Map No.	Region
Heilongjiang	Qiqihar	1	NSCR
Heilongjiang	Zhaodong	2	NSCR
Heilongjiang	Harbin	3	NSCR
Jilin	Songyuan	4	NSCR
Jilin	Nongan	5	NSCR
Jilin	Gongzhuling	6	NSCR
Inner Mongolia	Tongliao	7	NSCR
Liaoning	Tieling	8	NSCR
Liaoning	Dalian	9	NSCR
Shandong	Dezhou	10	HHHSCR
Henan	Xinxiang	11	HHHSCR
Henan	Zhengzhou	12	HHHSCR
Henan	Luoyang	13	HHHSCR
Anhui	Hefei	14	HHHSCR

NSCR: North Spring Corn Region; HHHSCR: Huang-Huai-Hai Summer Corn Region.

## Data Availability

Not applicable.

## Supplementary Materials

The following supporting information can be downloaded at: https://www.mdpi.com/article/10.3390/toxins15020137/s1, Table S1: Toxicity of Cry1Ab against *Ostrinia furnacalis* field populations in 2015–2021; Table S2: Toxicity of Cry1F against *Ostrinia furnacalis* field populations in 2015–2021.
