# Supplementary material for: Seven Years of Monitoring Susceptibility to Cry1Ab and Cry1F in Asian Corn Borer
Source: Toxins (Basel). 2023 Feb 7;15(2):137. doi: 10.3390/toxins15020137 (PMC9967349; doi:10.3390/toxins15020137)
Supplement: Supplementary file 1 [file toxins-15-00137-s001.zip › toxins-2155182-supplementary.pdf]

Table S1: Toxicity of Cry1Ab against *Ostrinia furnacalis* field populations in 2015-2021

Year	Population	n	LC_{50} (95% FL) μg/g	LC_{95} (95% FL) μg/g	RR (95% CI)	Slope ± SE	χ^2	*df* (χ^2)
2015								
	Of-S	624	0.28 (0.24 - 0.34)	3.73 (2.55 - 6.13)	1.00 (0.77 - 1.30)	1.47 ± 0.11	9.4	11
	Dezhou	576	0.33 (0.15 - 0.49)	2.31 (1.53 -5.49)	1.18 (0.73 -1.89)	1.95 ± 0.36	10.5	10
	Nongan	480	0.33 (0.24 - 0.44)	2.07 (1.48 -3.48)	1.18 (0.83 - 1.68)	2.07±0.28	6.5	8
	Songyuan	480	0.18 (0.12 - 0.22)	0.46 (0.34 - 1.34)	0.65 (0.50 - 0.83)	4.06 ± 0.87	10.9	8
	Zhengzhou	384	0.18 (0.15 - 0.22)	0.92 (0.69 - 1.36)	0.65 (0.50 - 0.84)	2.35 ± 0.24	4.8	6
	Xinxiang	384	0.18 (0.09 - 0.30)	3.39 (1.40 - 5.08)	0.63 (0.41 - 0.97)	1.28 ± 0.20	7.7	6
	Tieling	432	0.15 (0.12 - 0.18)	0.80 (0.61 - 1.16)	0.53 (0.40 - 0.71)	2.27 ± 0.24	3.2	7
	Tongliao	480	0.15 (0.12 - 0.18)	1.30 (0.92 - 2.08)	0.53 (0.39 - 0.71)	1.75 ± 0.17	4.0	8
	Dalian	672	0.11 (0.07 - 0.15)	1.16 (0.84 - 1.84)	0.39 (0.27 - 0.58)	1.62 ± 0.19	4.0	12
	Hefei	672	0.21 (0.15 - 0.26)	1.17 (0.89 - 1.77)	0.73 (0.53 - 1.01)	2.19 ± 0.27	7.4	12
	Gongzhuling	576	0.12 (0.08 - 0.17)	3.40 (2.15 - 6.23)	0.43 (0.29 - 0.65)	1.14 ± 0.10	4.5	10
2016								
	Of-S	672	0.22 (0.19 - 0.27)	1.28 (0.97 - 1.86)	1.00 (0.78 - 1.29)	2.17 ± 0.20	6.8	12
	Dezhou	624	0.27 (0.21 - 0.33)	1.85 (1.36 - 2.87)	1.19 (0.90 - 1.58)	1.96 ± 0.21	3.7	11
	Xinxiang	672	0.25 (0.20 - 0.30)	4.42 (2.98 - 7.32)	1.10 (0.84 - 1.43)	1.31 ± 0.09	6.9	12
	Zhaodong	672	0.22 (0.17 - 0.29)	5.70 (3.58 - 10.56)	0.99 (0.71 - 1.38)	1.17 ± 0.10	7.7	12
	Nongan	672	0.20 (0.16 - 0.25)	3.54 (2.40 - 5.86)	0.90 (0.66 - 1.22)	1.32 ± 0.10	7.3	12
	Songyuan	672	0.19 (0.16 - 0.23)	1.59 (1.19 - 2.31)	0.87 (0.67 - 1.12)	1.80 ± 0.14	6.2	12
	Gongzhuling	672	0.18 (0.14 - 0.23)	3.76 (2.48 - 6.51)	0.87 (0.67 - 1.12)	1.25 ± 0.10	4.1	12
	Qiqihar	624	0.17 (0.13 - 0.22)	2.33 (1.59 - 3.92)	0.76 (0.54 - 1.05)	1.44 ± 0.14	4.7	11
	Hefei	672	0.15 (0.11 - 0.18)	2.03 (1.44 - 3.12)	0.65 (0.48 - 0.87)	1.44 ± 0.11	7.1	12
	Tieling	672	0.12 (0.09 - 0.15)	2.79 (1.85 - 4.80)	0.52 (0.38 - 0.72)	1.19 ± 0.09	5.0	12
2017								
	Of-S	720	0.27 (0.21 - 0.34)	4.41 (3.14 - 6.78)	1.00 (0.72 - 1.39)	1.36 ± 0.10	9.2	13
	Dezhou	672	0.37 (0.25 - 0.50)	6.62 (4.14 - 13.07)	1.39 (0.90 - 2.13)	1.31 ± 0.14	7.4	12
	Zhaodong	768	0.31 (0.20 - 0.42)	3.81 (2.52 - 7.34)	1.16 (0.75 - 1.82)	1.51 ± 0.20	7.8	14
	Nongan	768	0.24 (0.16 - 0.32)	4.38 (2.84 - 8.29)	0.89 (0.57 - 1.38)	1.30 ± 0.15	7.5	14
	Qiqihar	672	0.23 (0.16 - 0.30)	3.35 (2.09 - 6.77)	0.85 (0.57 - 1.28)	1.41 ± 0.16	8.4	12
	Gongzhuling	672	0.21 (0.16 - 0.26)	2.25 (1.51 - 4.03)	0.78 (0.53 - 1.13)	1.58 ± 0.17	7.2	12
	Harbin	672	0.19 (0.14 - 0.26)	2.69 (1.85 - 4.47)	0.73 (0.48 - 1.12)	1.44 ± 0.14	4.1	12
	Tieling	672	0.17 (0.12 - 0.23)	3.26 (2.16 - 5.65)	0.64 (0.42 - 0.98)	1.29 ± 0.12	9.7	12
	Luoyang	576	0.16 (0.12 - 0.21)	2.49 (1.59 - 4.69)	0.60 (0.40 - 0.90)	1.38 ± 0.14	5.1	10
	Songyuan	672	0.15 (0.10 - 0.22)	4.22 (2.43 - 9.73)	0.58 (0.36 - 0.94)	1.15 ± 0.14	6.6	12
2018								
	Of-S	768	0.33 (0.25 - 0.42)	8.33 (5.49 - 14.25)	1.00 (0.70 - 1.44)	1.17 ± 0.09	8.3	14
	Dezhou	672	0.26 (0.20 - 0.33)	3.83 (2.57 - 6.52)	0.78 (0.55 - 1.11)	1.41 ± 0.12	8.4	12
	Luoyang	672	0.21 (0.15 - 0.28)	3.45 (2.32 - 5.98)	0.65 (0.43 - 0.94)	1.36 ± 0.13	8.8	12
	Gongzhuling	672	0.17 (0.14 - 0.20)	2.91 (2.02 - 4.66)	0.50 (0.37 - 0.70)	1.32 ± 0.09	6.4	12
	Harbin	768	0.16 (0.12 - 0.20)	2.85 (1.98 - 4.54)	0.49 (0.34 - 0.70)	1.32 ± 0.10	7.9	14

	Qiqihar	672	0.13 (0.10 - 0.17)	2.36 (1.63 - 3.81)	0.40 (0.28 - 0.57)	1.32 ± 0.10	7.6	12
	Songyuan	480	0.12 (0.09 - 0.15)	1.39 (0.91 - 2.56)	0.35 (0.25 - 0.50)	1.53 ± 0.16	5.9	8
	Zhaodong	528	0.11 (0.09 - 0.13)	0.77 (0.58 - 1.13)	0.32 (0.23 - 0.44)	1.91 ± 0.16	5.9	9
	Tieling	624	0.10 (0.08 - 0.13)	1.27 (0.90 - 1.98)	0.31 (0.21 - 0.45)	1.51 ± 0.13	7.6	11
	Nongan	672	0.08 (0.06 - 0.10)	1.39 (0.95 - 2.29)	0.24 (0.16 - 0.35)	1.31 ± 0.11	6.8	12
2019								
	Of-S	672	0.24 (0.17 - 0.31)	2.22 (1.60 - 3.57)	1.00 (0.67 - 1.50)	1.71 ± 0.19	6.3	12
	Zhaodong	672	0.09 (0.06 - 0.13)	1.33 (0.93 - 2.11)	0.39 (0.24 - 0.61)	1.43 ± 0.14	9.0	12
	Qiqihar	672	0.16 (0.12 - 0.20)	1.87 (1.36 - 2.84)	0.67 (0.46 - 0.97)	1.54 ± 0.12	9.2	12
	Harbin	672	0.17 (0.12 - 0.22)	1.64 (1.20 - 2.55)	0.70 (0.45 - 1.06)	1.66 ± 0.18	7.0	12
	Songyuan	672	0.07 (0.05 - 0.08)	0.79 (0.58 - 1.17)	0.27 (0.19 - 0.39)	1.52 ± 0.12	6.9	12
	Nongan	672	0.05 (0.03 - 0.07)	2.42 (1.47 - 4.81)	0.21 (0.13 - 0.34)	0.98 ± 0.09	6.0	12
	Gongzhuling	672	0.15 (0.11 - 0.19)	1.60 (1.17 - 2.41)	0.62 (0.42 - 0.91)	1.59 ± 0.14	8.1	12
	Tieling	672	0.14 (0.11 - 0.18)	2.90 (1.95 - 4.84)	0.59 (0.40 - 0.85)	1.25 ± 0.09	3.9	12
	Dezhou	672	0.23 (0.17 - 0.30)	2.03 (1.48 - 3.19)	0.97 (0.65 - 1.45)	1.75 ± 0.19	6.3	12
2020								
	Of-S	768	0.28 (0.20 - 0.36)	3.20 (2.36 - 4.72)	1.00 (0.67 - 1.50)	1.55 ± 0.13	5.2	14
	Zhaodong	768	0.22 (0.16 - 0.29)	4.10 (2.84 - 6.55)	0.80 (0.53 - 1.21)	1.30 ± 0.11	9.4	14
	Gongzhuling	768	0.20 (0.14 - 0.27)	2.53 (1.82 - 3.90)	0.72 (0.46 - 1.12)	1.49 ± 0.14	5.1	14
	Qiqihar	768	0.17 (0.13 - 0.21)	1.59 (1.21 - 2.27)	0.62 (0.43 - 0.90)	1.70 ± 0.14	5.6	14
	Harbin	768	0.15 (0.10 - 0.20)	1.28 (0.96 - 1.92)	0.53 (0.34 - 0.84)	1.75 ± 0.20	6.9	14
	Dezhou	768	0.13 (0.09 - 0.18)	1.62 (1.19 - 2.41)	0.48 (0.31 - 0.73)	1.52 ± 0.14	8.6	14
	Hefei	768	0.11 (0.08 - 0.14)	1.57 (1.13 - 2.37)	0.38 (0.25 - 0.58)	1.41 ± 0.11	7.9	14
2021								
	Of-S	768	0.28 (0.21 - 0.35)	2.16 (1.63 - 3.15)	1.00 (0.70 - 1.43)	1.86 ± 0.18	3.8	14
	Qiqihar	768	0.26 (0.19 - 0.32)	1.79 (1.37 - 2.59)	0.92 (0.64 - 1.32)	1.96 ± 0.20	8.7	14
	Harbin	768	0.20 (0.14 - 0.27)	3.16 (2.24 - 4.95)	0.71 (0.47 - 1.07)	1.37 ± 0.12	5.0	14
	Zhaodong	768	0.31 (0.25 - 0.38)	2.44 (1.85 - 3.51)	1.11 (0.79 - 1.55)	1.84 ± 0.16	8.4	14
	Gongzhuling	768	0.23 (0.14 - 0.33)	4.37 (2.96 - 7.43)	0.82 (0.50 - 1.33)	1.29 ± 0.14	4.1	14
	Dezhou	768	0.19 (0.13 - 0.26)	2.30 (1.67 - 3.52)	0.68 (0.45 - 1.04)	1.53 ± 0.15	6.4	14

Table S2: Toxicity of Cry1F against *Ostrinia furnacalis* field populations in 2015-2021

Year	Population	n	LC_{50} (95% FL) μg/g	LC_{95} (95% FL) μg/g	RR (95% CI)	Slope±SE	χ^2	df (χ^2)
2015								
	Of-S	672	0.93 (0.74 - 1.15)	23.99(15.73- 41.55)	1.00 (0.73 - 1.37)	1.17 ± 0.09	6.5	12
	Gongzhuling	576	1.16 (0.65 - 1.68)	12.62 (8.97 - 22.72)	1.25 (0.76 - 2.07)	1.57 ± 0.22	7.8	10
	Dalian	576	1.06 (0.78 - 1.35)	7.24 (5.22 - 11.65)	1.14 (0.81 - 1.61)	1.97 ± 0.23	7.9	10
	Xinxiang	480	1.22 (0.87 - 1.59)	8.11 (5.23 - 17.47)	1.31 (0.91 - 1.89)	1.99 ± 0.31	6.0	8
	Dezhou	576	0.54 (0.31 - 0.78)	7.04 (4.78 - 12.39)	0.58 (0.35 - 0.94)	1.47 ± 0.19	9.3	10
	Songyuan	432	0.73 (0.53 - 0.98)	3.75 (2.28 - 10.52)	0.78 (0.54 - 1.12)	2.31 ± 0.45	6.1	7
	Hefei	576	0.44 (0.32 - 0.58)	4.39 (3.07 - 7.26)	0.48 (0.33 - 0.69)	1.65 ± 0.18	3.8	10
	Nongan	576	0.52 (0.38 - 0.67)	2.47 (1.79 - 4.00)	0.56 (0.39 - 0.80)	2.43 ± 0.32	9.8	10
	Tieling	576	0.32 (0.23 - 0.42)	3.52 (2.43 - 5.89)	0.34 (0.23 - 0.50)	1.58 ± 0.16	4.8	10
	Zhengzhou	528	0.19 (0.14 - 0.24)	1.77 (1.27 - 2.78)	0.20 (0.14 - 0.29)	1.69 ± 0.16	3.8	9
	Tongliao	624	0.14 (0.10 - 0.17)	1.57 (1.12 - 2.46)	0.15 (0.10 - 0.21)	1.55 ± 0.13	4.2	11
2016								
	Of-S	624	0.39 (0.30 - 0.49)	5.59 (3.84 - 9.19)	1.00 (0.71 - 1.41)	1.42 ± 0.12	4.0	11
	Dezhou	624	0.24 (0.18 - 0.31)	3.45 (2.36 - 5.78)	0.63 (0.43 - 0.90)	1.43 ± 0.13	2.4	11
	Gongzhuling	576	0.22 (0.17 - 0.27)	4.15 (2.79 - 6.96)	0.56 (0.40 - 0.78)	1.52 ± 0.14	2.5	10
	Nongan	672	0.21 (0.16 - 0.27)	4.15 (2.79 - 6.96)	0.54 (0.37 - 0.78)	1.27 ± 0.10	4.2	12
	Zhaodong	624	0.21 (0.16 - 0.27)	2.31 (1.63 - 3.68)	0.54 (0.37 - 0.77)	1.58 ± 0.15	5.5	11
	Xinxiang	624	0.17 (0.13 - 0.21)	3.50 (2.32 - 6.02)	0.43 (0.31 - 0.59)	1.24 ± 0.10	9.6	11
	Tieling	576	0.16 (0.11 - 0.21)	1.54 (1.07 - 2.67)	0.41 (0.28 - 0.60)	1.67 ± 0.21	5.9	10
	Hefei	576	0.11 (0.09 - 0.15)	1.57 (1.09 - 2.53)	0.29 (0.20 - 0.42)	1.44 ± 0.13	8.5	10
	Qiqihar	576	0.11 (0.08 - 0.14)	2.45 (1.53 - 4.73)	0.28 (0.19 - 0.41)	1.21 ± 0.12	4.1	10
	Songyuan	672	0.10 (0.08 - 0.13)	4.15 (2.79 - 6.96)	0.27 (0.19 - 0.37)	1.51 ± 0.11	9.9	12
2017								
	Of-S	672	0.25 (0.20 - 0.32)	3.41 (2.42 - 5.35)	1.00 (0.70 - 1.42)	1.46 ± 0.12	5.6	12
	Zhaodong	768	0.27 (0.19 - 0.37)	3.39 (2.42 - 5.33)	1.08 (0.72 - 1.63)	1.51 ± 0.15	7.8	14
	Luoyang	672	0.24 (0.17 - 0.31)	2.84 (1.92 - 5.11)	0.94 (0.63 - 1.40)	1.53 ± 0.18	8.2	12
	Dezhou	672	0.23 (0.16 - 0.31)	4.69 (3.03 - 8.48)	0.92 (0.61 - 1.38)	1.26 ± 0.12	5.3	12
	Nongan	624	0.23 (0.16 - 0.30)	2.69 (1.84 - 4.69)	0.92 (0.62 - 1.37)	1.54 ± 0.17	7.3	11
	Songyuan	672	0.22 (0.15 - 0.29)	2.98 (1.90 - 6.05)	0.87 (0.58 - 1.31)	1.46 ± 0.19	4.8	12
	Gongzhuling	768	0.20 (0.14 - 0.28)	5.24 (3.29 - 10.01)	0.80 (0.52 - 1.24)	1.17 ± 0.12	6.4	14
	Harbin	768	0.16 (0.11 - 0.22)	4.09 (2.73 - 6.89)	0.64 (0.42 - 0.98)	1.17 ± 0.10	5.5	14
	Qiqihar	672	0.14 (0.10 - 0.20)	4.51 (2.68 - 9.25)	0.56 (0.36 - 0.87)	1.09 ± 0.11	7.4	12
	Tieling	672	0.11 (0.07 - 0.14)	1.06 (0.76 - 1.73)	0.42 (0.28 - 0.63)	1.65 ± 0.19	11.1	12
2018								
	Of-S	672	0.92 (0.73 - 1.12)	9.30 (6.51 - 15.14)	1.00 (0.74 - 1.35)	1.63 ± 0.15	5.4	12
	Dezhou	672	0.68 (0.54 - 0.84)	6.94 (4.80 - 11.56)	0.74 (0.55 - 1.01)	1.63 ± 0.15	5.9	12
	Luoyang	672	0.54 (0.44 - 0.66)	5.22 (3.59 - 8.75)	0.59 (0.44 - 0.79)	1.67 ± 0.15	6.4	12
	Gongzhuling	672	0.30 (0.25 - 0.35)	2.91 (2.14 - 4.28)	0.33 (0.25 - 0.43)	1.66 ± 0.11	8.5	12
	Qiqihar	672	0.28 (0.22 - 0.33)	2.11 (1.58 - 3.09)	0.30 (0.23 - 0.40)	1.86 ± 0.16	7.9	12

	Harbin	624	0.25 (0.19 - 0.30)	1.84 (1.35 - 2.85)	0.27 (0.20 - 0.37)	1.88 ± 0.20	8.4	11
	Nongan	720	0.23 (0.16 - 0.31)	5.95 (3.91 - 10.23)	0.25 (0.17 - 0.37)	1.16 ± 0.10	2.5	13
	Tieling	672	0.21 (0.16 - 0.27)	2.67 (1.93 - 4.05)	0.23 (0.17 - 0.32)	1.49 ± 0.12	7.9	12
	Songyuan	576	0.18 (0.14 - 0.23)	2.15 (1.52 - 3.44)	0.20 (0.14 - 0.28)	1.53 ± 0.14	5.3	10
	Zhaodong	576	0.18 (0.14 - 0.23)	3.57 (2.31 - 6.51)	0.20 (0.14 - 0.28)	1.27 ± 0.12	7.2	10
2019								
	Of-S	768	0.53 (0.40 - 0.68)	11.56 (7.49 - 20.65)	1.00 (0.69 - 1.45)	1.23 ± 0.12	5.4	14
	Zhaodong	672	0.20 (0.14 - 0.26)	2.53 (1.77 - 4.09)	0.37 (0.25 - 0.55)	1.48 ± 0.14	2.5	12
	Qiqihar	672	0.18 (0.15 - 0.22)	1.71 (1.27 - 2.51)	0.34 (0.24 - 0.48)	1.69 ± 0.13	8.3	12
	Harbin	672	0.26 (0.20 - 0.32)	2.72 (1.95 - 4.22)	0.49 (0.34 - 0.69)	1.61 ± 0.14	7.6	12
	Songyuan	672	0.20 (0.15 - 0.24)	1.29 (0.99 - 1.84)	0.37 (0.26 - 0.52)	2.01 ± 0.19	5.6	12
	Nongan	672	0.15 (0.12 - 0.18)	1.28 (0.97 - 1.83)	0.28 (0.20 - 0.39)	1.75 ± 0.13	9.7	12
	Gongzhuling	672	0.11 (0.09 - 0.13)	0.55 (0.44 - 0.74)	0.21 (0.16 - 0.29)	2.38 ± 0.18	9.0	12
	Tieling	672	0.17 (0.13 - 0.21)	2.48 (1.74 - 3.90)	0.32 (0.23 - 0.45)	1.41 ± 0.10	2.4	12
	Dezhou	672	0.26 (0.19 - 0.33)	3.23 (2.25 - 5.34)	0.49 (0.34 - 0.72)	1.50 ± 0.15	8.2	12
2020								
	Of-S	864	0.52 (0.36 - 0.70)	8.96 (6.37 - 13.86)	1.00 (0.63 - 1.59)	1.33 ± 0.11	6.4	16
	Zhaodong	768	0.58 (0.42 - 0.75)	9.36 (6.55 - 14.88)	1.11 (0.72 - 1.71)	1.36 ± 0.11	6.7	14
	Gongzhuling	768	0.45 (0.29 - 0.60)	3.69 (2.73 - 5.68)	0.86 (0.53 - 1.40)	1.80 ± 0.22	8.1	14
	Qiqihar	768	0.52 (0.43 - 0.63)	4.99 (3.78 - 7.08）	1.00 (0.68 - 1.47)	1.67 ± 0.12	5.3	14
	Harbin	768	0.44 (0.30 - 0.61)	10.81 (7.17 - 18.47)	0.86 (0.53 - 1.39)	1.19 ± 0.10	4.3	14
	Dezhou	768	0.37 (0.28 - 0.47)	4.71 (3.44 - 6.99)	0.72 (0.47 - 1.09)	1.49 ± 0.12	3.2	14
	Hefei	768	0.28 (0.21 - 0.37)	4.66 (3.32 - 7.13)	0.55 (0.35 - 0.85)	1.35 ± 0.11	7.9	14
2021								
	Of-S	768	0.63 (0.46 - 0.81)	7.40 (5.43 - 10.98)	1.00 (0.67 - 1.49)	1.53 ± 0.13	5.6	14
	Qiqihar	864	0.58 (0.41 - 0.76)	5.15 (3.84 - 7.66)	0.93 (0.61 - 1.40)	1.73 ± 0.18	7.2	16
	Harbin	864	0.34 (0.24 - 0.44)	4.52 (3.32 - 6.68)	0.54 (0.35 - 0.81)	1.46 ± 0.12	6.2	16
	Zhaodong	768	0.45 (0.31 - 0.60)	5.95 (4.27 - 9.27)	0.72 (0.46 - 1.10)	1.47 ± 0.14	6.7	14
	Gongzhuling	864	0.44 (0.31 - 0.58)	4.63 (3.44 - 6.83)	0.70 (0.46 - 1.08)	1.61 ± 0.15	9.1	16
	Dezhou	768	0.25 (0.17 - 0.33)	5.04 (3.48 - 8.07)	0.39 (0.25 - 0.61)	1.25 ± 0.10	5.9	14
